# Serology reveals micro-differences in *Plasmodium falciparum* transmission in the Hohoe municipality of Ghana

**DOI:** 10.3389/fpara.2023.1081083

**Published:** 2023-01-27

**Authors:** Eric Kyei-Baafour, Kwadwo A. Kusi, Mavis Oppong, Abena F. Frempong, Belinda Aculley, Ebenezer A. Ofori, Michael Theisen, Margaret Kweku, Bright Adu, Lars Hviid, Michael F. Ofori

**Affiliations:** ^1^ Department of Immunology, Noguchi Memorial Institute for Medical Research, College of Health Sciences, University of Ghana, Accra, Ghana; ^2^ Department of Epidemiology and Biostatistics, School of Public Health, University of Health and Allied Sciences, Hohoe, Ghana; ^3^ Centre for Medical Parasitology, Department of Immunology and Microbiology, University of Copenhagen, Copenhagen, Denmark; ^4^ Department for Congenital Disorders, Statens Serum Institute, Copenhagen, Denmark; ^5^ Department of Infectious Diseases, Rigshospitalet, Copenhagen, Denmark

**Keywords:** malaria, serology, *Plasmodium falciparum*, micro-differences, transmission, immunoglobulin G

## Abstract

**Background:**

With the decline in malaria transmission due to global efforts, a more sensitive tool is needed to monitor transmission intensity and pattern at the micro-level. Though transmission in a broader area may be similar, factors such as sanitation, practices of open water storage, early morning and evening activities, outdoor sleeping and agricultural practices within communities could cause differences in exposure and thus transmission. This study thus probed malaria transmission at a micro-level using serology in the Hohoe Municipality of Ghana.

**Methods:**

This cross-sectional study involved 327 asymptomatic children aged 1-12 years in both rural (196) and urban (131) communities in the Hohoe municipality. Total IgG responses specific for three P. falciparum antigens (CSP, MSP2-FC27, MSP2-3D7) were determined in plasma eluted from dried blood spots using indirect ELISA.

**Results:**

A higher proportion of individuals in the rural area had parasites by both microscopy and PCR. Total IgG levels and seroprevalence were higher in rural compared to urban communities (p<0.05). In a multiple regression model, adjusting for confounders, levels of PfMSP2-3D7-specific IgG was associated with the higher transmission which occurs in the rural community.

**Conclusion:**

The results suggest that though the district is categorized as having medium malaria transmission, differences within settlements may influence malaria transmission reflecting in antibody levels and prevalence of malaria antigen-specific IgG.

## Introduction

Malaria caused by *Plasmodium falciparum* is a major health care challenge in sub-Saharan Africa (SSA). The burden of malaria is high with an estimated 94% of all malaria deaths occurring in SSA (WHO, 2020). Over the last two decades, local transmission of malaria in some countries in Africa is declining, and child mortality has also dropped as a result of the control measures adopted by governments (Campbell and Steketee, 2011).

In Ghana, about 30% of out-patients in health facilities have malaria (NMCP, 2016). Malaria transmission in Ghana is seasonal, varying from high in the rainy season to low in the dry season. Malaria transmission also differs according to the three ecological zones of Ghana, being low in the coastal scrub, medium in the semi-deciduous and transitional forest, and high in the guinea savanna zone (Afari et al., 1992).

The conventional tools used in estimating malaria transmission intensity are entomological inoculation rates (EIR) based on human landing catches and parasite prevalence (Hay et al., 2000; Corran et al., 2007). Alternative methods such as serology (antibody responses to vector-specific or parasite-specific antigens) have been proposed and tested to be robust and sensitive (Corran et al., 2007; Badu et al., 2012; Kusi et al., 2014; Badu et al., 2015). Serology can thus reveal micro-differences in a larger transmission zone, especially those with medium to low transmission. Local parasite transmission is dependent on environmental factors, land use, human settlements and behaviour, which may influence vector abundance and thus parasite transmission patterns (Yankson et al., 2019).

*Plasmodium falciparum* merozoite surface protein 2 (*Pf*MSP2) is a glycoprotein which is encoded by the MSP2 gene on chromosome 2. It is made up of five blocks with the central block being the most polymorphic. MSP2 is one of the numerous malaria vaccine candidates under consideration for a vaccine (Howard and Pasloske, 1993). However, it exhibits extensive polymorphism and the genes of MSP2 in the block 3 regions of MSP2 have alleles grouped into two families, the IC3D7 and FC27 (Smythe et al., 1991). These have been used to determine the multiplicity of *P. falciparum* infection (Felger et al., 1994) which is an indication of the diversity of the circulation parasites in an area. *Plasmodium falciparum* circumsporozoite protein (CSP) is a protein expressed extensively on sporozoite surfaces (Ménard et al., 1997), and exposed to the immune system for a brief time after an infectious bite. Meaning anti-CSP antibodies may have a relatively shorter half-life (Kerkhof et al., 2016) than most blood-stage antigens, and thus positive responses may indicate a recent infection. With the recent decline in malaria transmission, it is becoming increasingly difficult to use the conventional entomological inoculation rate (EIR) or parasite prevalence to estimate transmission intensity due to the very low prevalence of infected mosquitoes, thus proposing serology as an alternative (Corran et al., 2007; Rosas-Aguirre et al., 2013). For example, anti-CSP antibodies have been found to be ideal for monitoring malaria transmission intensity and pattern (Kusi et al., 2014). We recently reported on the suitability of multiple malaria antigens to estimate transmission intensity and pattern in three ecological zones in the Volta Region of Ghana (Kyei-Baafour et al., 2021). However, the local differences in transmission intensity and pattern within each ecological zone were not highlighted. Here, we report on the micro-differences in *P. falciparum* transmission within a medium malaria transmission zone in the Hohoe Municipality.

## Methods

### Population and study sites

This study involving a total of 327 children aged 1-12 years, was a cross-sectional community-based survey carried out in the Hohoe Municipality of the Volta region of Ghana. Sampling for the study was carried out in December 2018 which was in the dry season. The Hohoe municipality has two major seasons: a rainy season which spans the months of May and October, and a dry season which starts from November and ends in April. The rainfall pattern in the areas around Hohoe is bimodal which peaks in June. There is also a minor peak in October (Ofori et al., 2018)

The Research Ethics Committee of the University of Health and Allied Sciences (UHAS-REC A.1 (5) 18–19) approved the study. Written informed consent was obtained from parents/guardians of the children before sampling. All children sampled had no clinical symptoms. The population and sites of the larger study have been described in detail elsewhere (Oppong et al., 2020; Kyei-Baafour et al., 2021).

**Figure 1:**
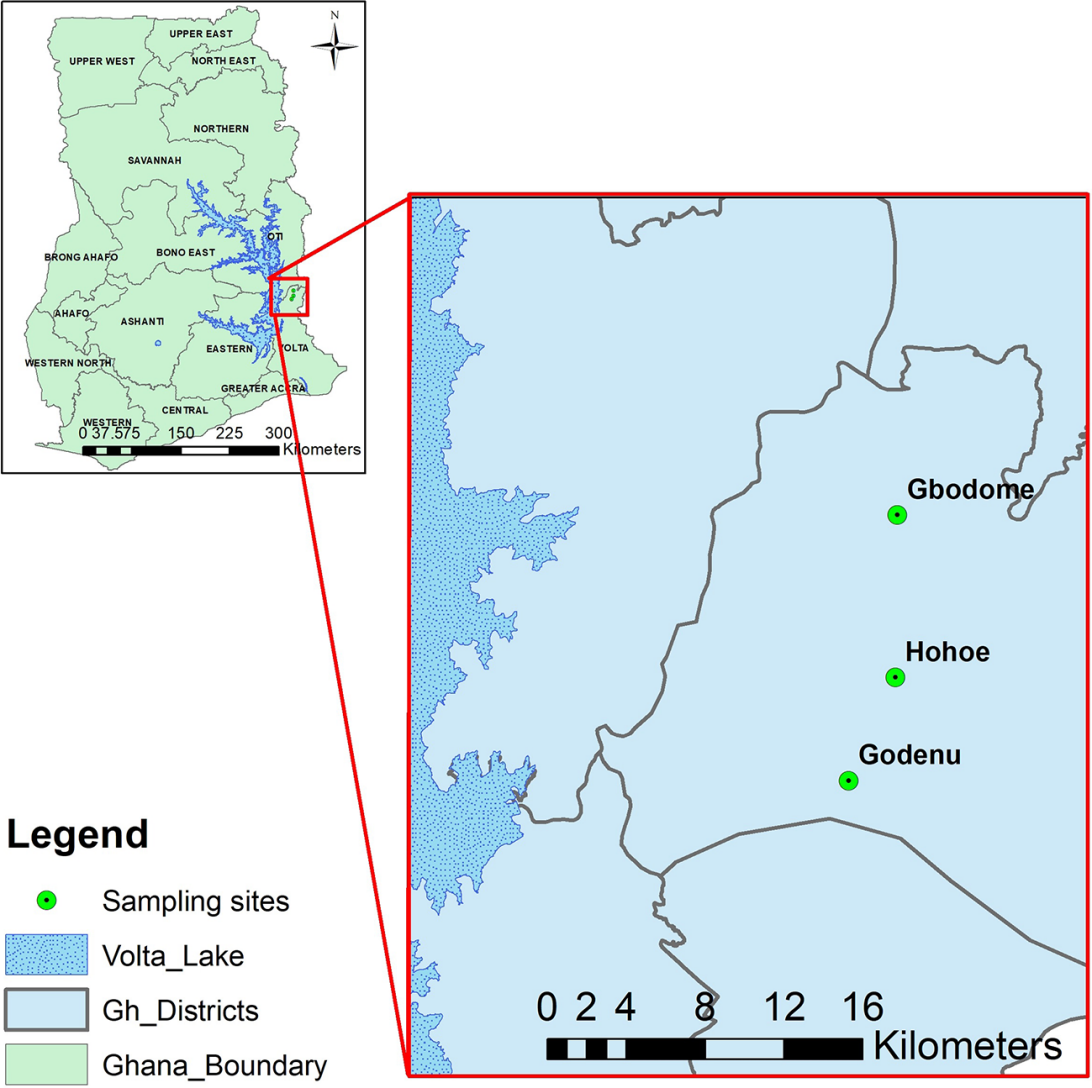
A map of the study area showing the sampling sites.The figure shows a map of the sampling sites with Hohoe the capital of the municipality (Urban) and the two rural settlements. The Map was created by Dr Prince Asare of the Noguchi Memorial Institute for Medical Research, University of Ghana, using QGIS version 3.4.7.

Briefly, the Hohoe Municipality has a population of about 114,472 out of which 52.6% are urban settlers. The municipality is in a medium transmission zone with a reported malaria prevalence of 7.7% (Kweku et al., 2017). More than 57% of the rural settlers engage in agriculture. The study sites included Hohoe, the urban settlement which is a commercial town with the biggest market in the municipality and connected with piped water and also hosts the municipal hospital. Godenu and Gbodome were the rural settlements studied, with Godenu located south of, and Gbodome north of Hohoe. The distance between Hohoe and Godenu is about 7km, and from Hohoe to Gbodome is about 9km (**Figure 1**).

### Sampling and elution of antibodies from dried blood spots

Blood spots from finger pricks were collected on Whatman No. 5 filter paper (GE Healthcare, England) and air-dried. The dried blood spots (DBS) were stored in desiccated bags at 4°C until ready for use. Plasma was eluted from the DBS using a protocol described previously (Corran et al., 2008). Briefly, individually wrapped filter papers were cut to 2.5 mm diameter with a leather punch into 96-well round bottom plates. Serum was eluted with 150 μL PBS with 0.05% Tween 20 and 0.05% sodium azide overnight. Eluted serum was stored at -20°C for later use.

### Determination of total IgG levels

Antigens selected for this study were expressed in a *Lactococcus lactis* expression system described previously (Singh et al., 2018). MSP2-3D7 and CSP were cloned from *P. falciparum* strains of 3D7 while MSP2-FVO, was cloned from *P. falciparum* FVO strain. The MSP2 antigens were selected due on their use as molecular markers for the determination of the multiplicity of *P. falciparum* infection (genetic diversity of parasite population) (Zhong et al., 2018; Mohammed et al., 2021) thus, this study was to test their suitability as serological markers of malaria transmission intensity estimation. The CSP was used as a control antigen since studies have found it as a useful marker in determining the heterogeneity of malaria transmission intensity and pattern (Kusi et al., 2014; Kusi et al., 2016).

Levels of IgG specific for each of the three *Plasmodium falciparum* antigens (MSP2-3D7, MSP2-FC27, and CSP) were determined using indirect ELISA. Briefly, 96-well microtiter plates (Maxisorp Nunc) were coated with 100 µL/well of 1 µg/mL of antigen in 1× PBS, pH of 7.4. The plates were incubated overnight at 4°C. Plates were blocked with 5% skimmed milk/PBS and incubated in a humidified chamber at room temperature for 1 h. The plates were incubated with 100 µL/well-eluted plasma samples and control samples (plasma from 10 naïve individuals who have never been to a malaria endemic area), all diluted 200× in 3% milk/PBS, for 1 hr. Plate development was done with 100 µL/well horseradish peroxidase (HRP) conjugated goat-anti-human IgG (H+L) (Life Technologies, USA) and incubated for 1 h. Antibody quantification was done using TMB substrate for 10 min in the dark and colour development stopped by the addition of 100 µL/well of 0.2 N H_2_SO_4_. Optical density (OD) was read at 450 nm. Optical density was converted to antibody unit (AU).

### Data analysis

Antibody units from the naïve volunteers were used to define a cut-off for seropositivity. the cut-off for seropositivity was calculated as the mean naïve antibody units plus 2 standard deviations of the mean. For each antigen, median antibody levels and seroprevalence were compared among the settlements using the Mann-Whitney test, and chi-square tests respectively. Six samples positive for both *P. falciparum* and *P. malariae* were excluded from the analysis. Associations between each antibody response and settlements were derived from a multivariate logistic regression model, adjusting for confounders and other antigens. Differences were considered statistically significant when p-values <0.05. Data were analyzed using R statistical software version 4.1.2, and figures made with GraphPad Prism version 9.0.0 (121).

## Results

### Study population

Children from both communities have similar ages, and the proportion of males to females was not statistically different. Bed net usage was also similar between the communities. They also had similar haemoglobin levels. Parasite density between the communities was not different. However, parasite prevalence by microscopy was 3.8% in the urban settlement compared to 9.7% in the rural community p=0.077. A similar trend was observed when prevalence was compared by PCR was between the two communities (7.6% vs 17.3%, p=0.018) for urban versus rural communities (**Table 1**)

**Table 1:** Study participant demographics.

Variable	Urban (n=131)	Rural (n=196)	Total (n=327)	p-value
**Age [mean years (SD)]**	5.4 (3.6)	5.6 (3.1)	5.6 (3.3)	0.618*
**Gender**				
Female	75 (57.3)	106 (54.1)	181 (55.4)	0.651^$^
Bed net usage (%)				
Yes	109 (83.2)	173 (88.3)	282 (86.2)	0.255^$^
**Parasite density [median (IQR)]**	960 (320 to 1224)	720 (440 to 3340)	780 (350 to 3190)	0.696^#^
Microscopy [N (%)]				
Positive	5 (3.8)	19 (9.7)	24 (7.3)	0.075^$^
**PCR [N (%)]**				
Positive	10 (7.6)	34 (17.3)	44 (13.5)	0.018^$^
**Hb/g/dl [mean (sd)]**	11.1 (1.6)	11.3 (1.8)	11.2 (1.7)	0.328*

*P value derived using students t-test, $ p value derived using chi-square test, # p value derived using Mann Whitney test.

### Parasitaemia and total IgG levels

**Figure 2:**
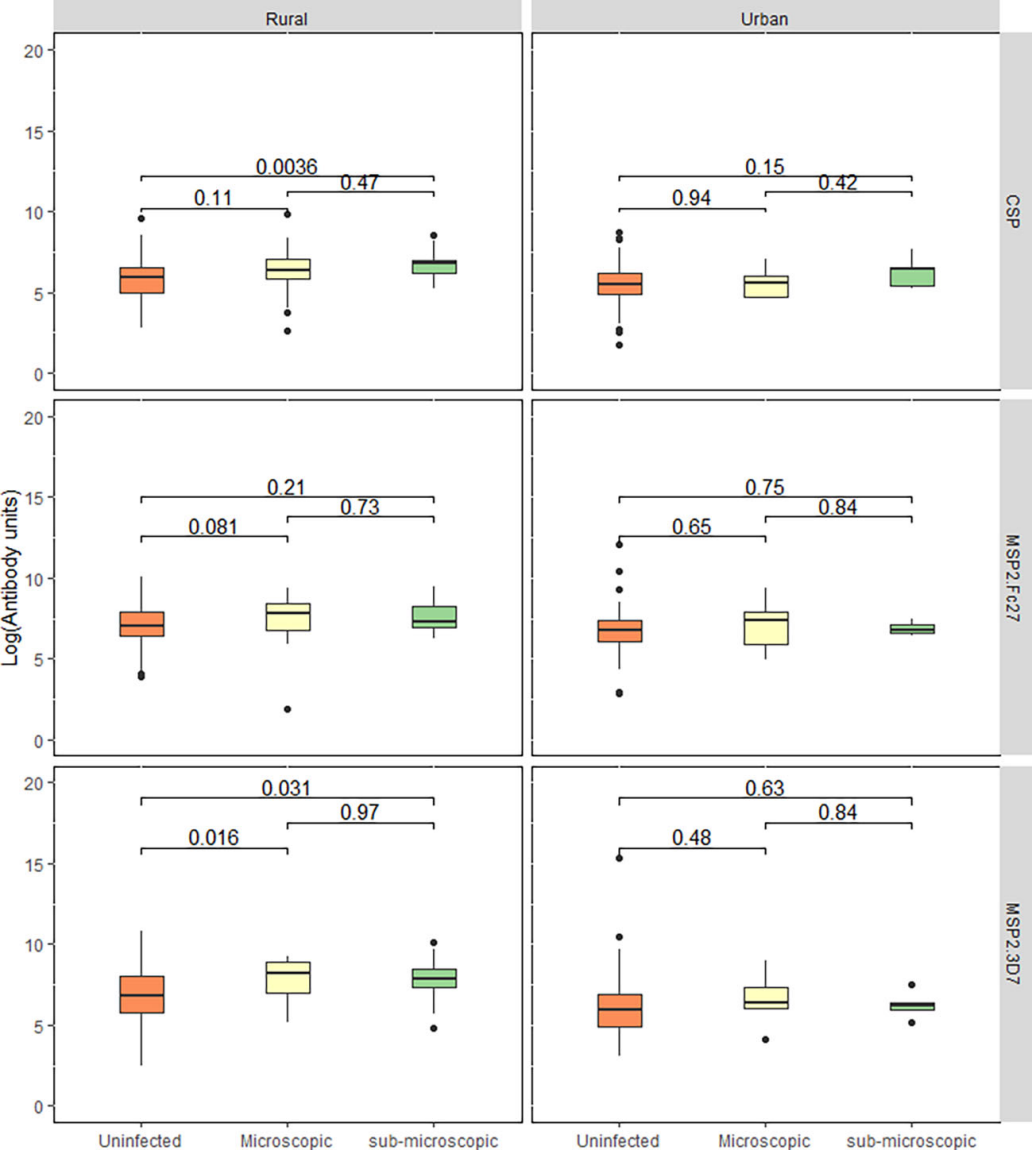
Total IgG response between parasitaemic and aparasitaemic children. The box and whisker plot represent log-transformed median IgG levels of children with uninfected, microscopic, and sub-microscopic parasitaemia for each antigen for both the rural and urban settlements. Central lines: medians, Boxes: central 50%, Whiskers: central 90%. Outliers are shown as dots. Kruskal-Wallis test was used to test for overall intergroup differences followed by Dunn’s post-hoc test, when overall intergroup statistical significance (p < 0.05).

Since the proportion of individuals positive for parasitaemia was higher in the rural community, we compared antibody levels between the three different parasite categories (microscopic, sub-microscopic, and those without detectable parasitaemia) in each community. In the urban community, no differences were observed among the three categories. However, those with sub-microscopic parasitemia had higher IgG levels to *Pf*CSP and *Pf*MSP2-3D7 in the rural community (p<0.05 for the two antigens, Kruskal Wallis test. **Figure 2**).

### Seroprevalence of *P. falciparum antigen*-specific antibodies

**Figure 3:**
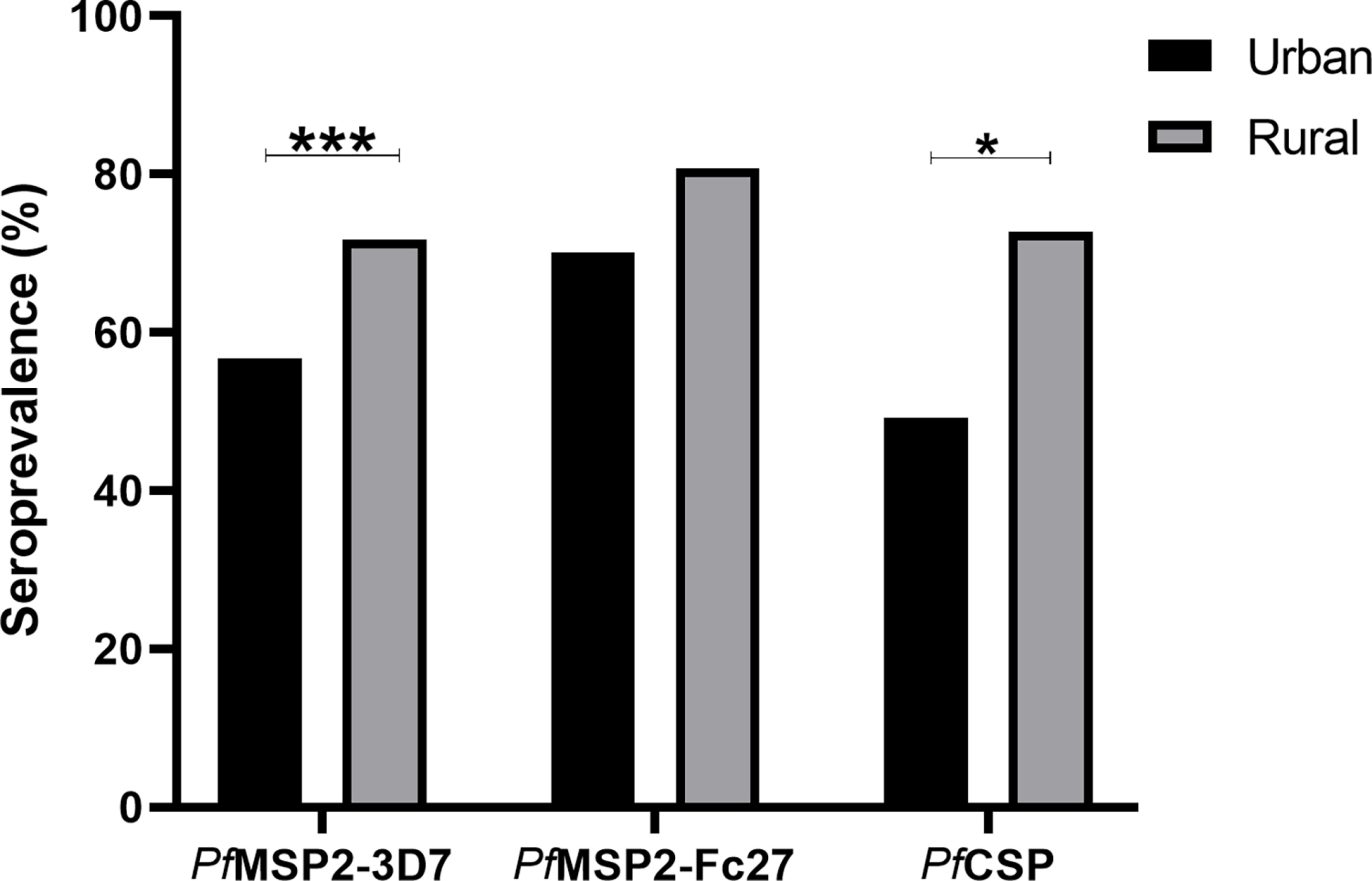
Seroprevalence was compared between the urban (black bars), and rural (grey bars) communities. P-values between communities were determined using the Chi-square test and *p = <0.05, *** p = <0.001.

Antibody seroprevalence according to the study communities was analyzed to explore the effects of the different settlements on antigen recognition. Antibody seroprevalence to *Pf*CSP (X^2 =^ 4.91. p = 0.03), and *Pf*MSP2-3D7 (X^2^ = 12.11, p=0.0005) but not *Pf*MSP2*-*Fc27 were significantly higher in the rural settlements (**Figure 3**).

Next, we explored and compared the age-specific seroprevalence between the two settlements. The children were categorized into three different age groups: 0-4 years, 5-8 years, and 9-12 years. In the rural settlements, children above nine (9) years had significantly higher antibody seroprevalence to all the antigens (p= 0.009 for *Pf*CSP; p<0.0001 for *Pf*MSP2-Fc27; and p<0.0001 for *Pf*MSP2-3D7). A similar trend was observed in the urban community for *Pf*CSP (p<0.0001), where children above 9 years had significantly higher seroprevalence. However, seroprevalence to *Pf*MSP2-Fc27 was higher in the 5-8-year group (84.1%, x^2 =^ 13.3 p=0.003), and responses to *Pf*MSP2-3D7 showed no difference among the age groups (**Table 2**)

**Table 2:** Age-related seroprevalence in rural and urban children.

Urban			
	*Pf*CSP	*Pf*MSP2-Fc27	*Pf*MSP2-3D7
0-4yrs	45.6	72.7	70
5-8yrs	77.2	84.1	76.7
9-12yrs	84.2	61.4	63.3
	X2 = 38.2 p<0.0001	x2 = 13.3 p=0.003	X2 = 4.7 p=0.97
Rural			
0-4yrs	67.9	66.7	56.4
5-8yrs	69.2	88.5	83.3
9-12yrs	84.6	94.9	84.6
	X2 = 9.5 p= 0.009	X2 = 30.7 p<0.0001	X2 = 27.7 p<0.0001

P values were generated using a chi-square test.

### Levels of IgG specific to Antibodies

**Figure 4:**
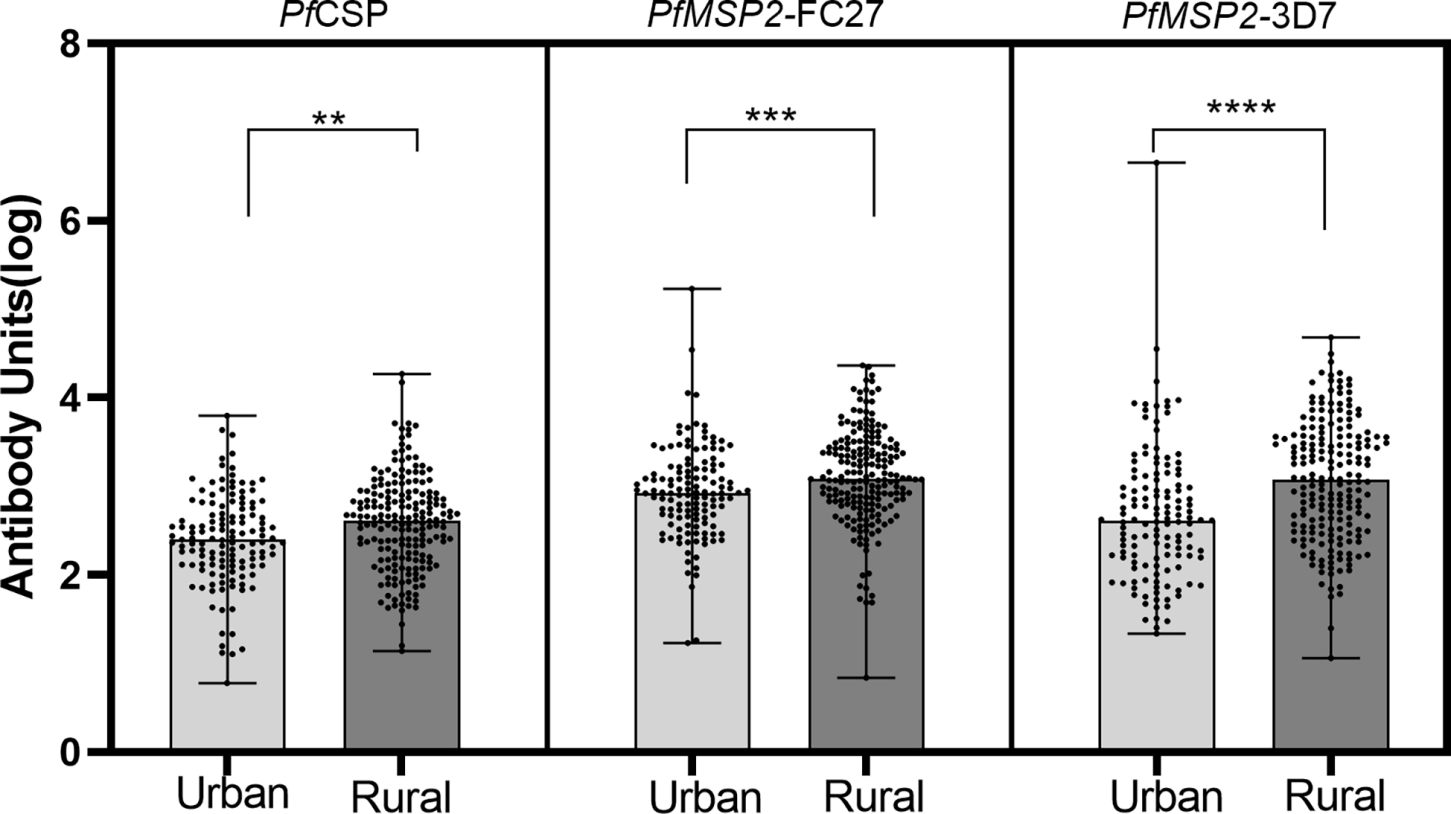
Median total IgG levels between urban and rural settlements. Log transformed antibody responses were determined using ELISA and are shown as a bar with dot plots. The boxes represent the median with error bars showing the upper and lower values. The dots show the distribution of individual antibody levels. Stars represent significantly higher antigens levels between the communities using the Mann-Whitney test. **p<0.01, ***p<0.001, ****p<0.0001.

We analyzed the total IgG levels to the three antigens in the rural and urban communities and found that median levels to all the three antigens were higher in the rural community compared to the urban community (p< 0.05; Mann–Whitney test, for all three antigens) (**Figure 4**).

**Figure 5:**
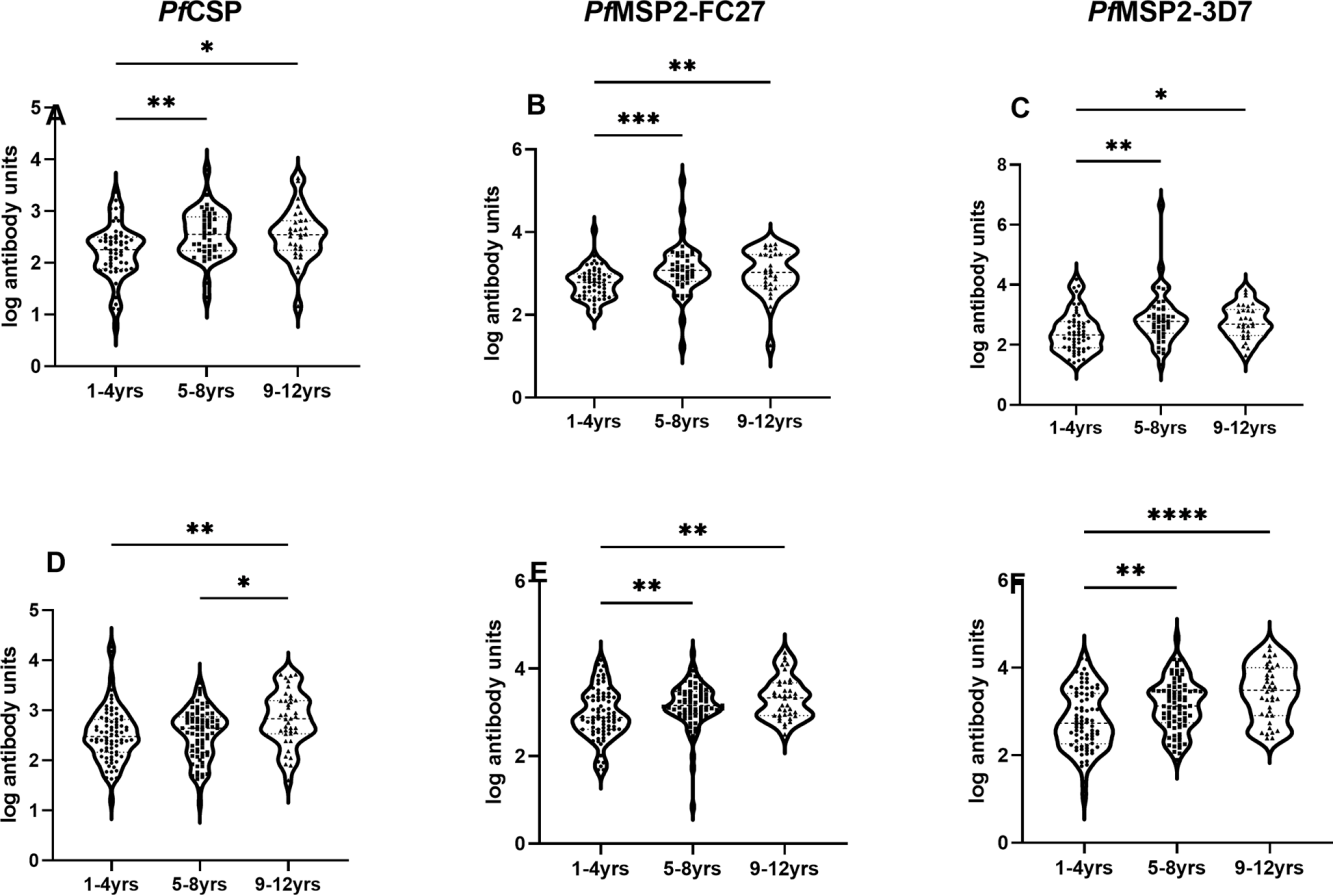
Association of antibody levels with age. Box plot of antibody units (log-transformed) and categorized ages for urban **(A-C)**, and rural **(D-F)** settlements. The error bars represent the minimum and maximum antibody units with the dots representing individual samples in each category. Stars represent significantly higher antigens levels among the three age categories using the Kruskal-Wallis test. *p = <0.05, **p<0.01, ***p<0.001, ****p<0.0001.

Levels of total IgG were compared among the three age groups and found children 9 years and above had significantly higher total IgG levels to all the antigens within each community compared to those under 5 years (p<0.05 for all antigens, **Figure 5**).

### Association between higher IgG responses and transmission

To determine whether malaria transmission could be predicted using antibody responses, the responses to each of the three antigens were individually analyzed using a multivariate logistic regression model with community of residence as a binary outcome variable and with antibody responses as the predictor variable. For the responses to each antigen, the models were adjusted for sub-microscopic parasitaemia, bed net usage and responses to other antigens. Our data showed that higher responses to MSP2-3D7 increased the odds of having higher malaria transmission in the rural community (OR= 1.37, 95%CI=1.15-1.64, p=0.0006) (**Table 3**).

**Table 3:** Association between IgG and Transmission.

Antigen	OR	95% CI	p-value
*Pf*MSP2-3D7	1.37	1.15 to 1.64	0.0006
*Pf*MSP2-Fc27	1.03	0.82 to1.29	0.81
*Pf*CSP	1.09	0.87 to 1.36	0.44

## Discussion

With the prevalence and transmission of malaria declining, sensitive tools are needed to effectively monitor transmission intensity and pattern at the micro-level. Serology is one key tool used to estimate transmission intensity and pattern because it can detect heterogeneity in transmission intensity at the micro level using antibody responses to malaria antigens.

We set out to determine the association between parasite prevalence and antibody responses to two *P. falciparum* merozoite antigens (MSP2-3D7, MSP2-FC27) and a sporozoite antigen (CSP) within different settlements of the same transmission zone. Our data indicate that the parasite prevalence was higher in rural settlements compared to urban settlements. Also, antibody responses to all antigens were higher in the rural settlements, and antibody seroprevalence to MSP2-3D7, and CSP were also higher in the rural settlements. In a multivariate analysis, we also found responses to *Pf*MSP2-3D7 were associated with high transmission and thus rural settlement. This is consistent with studies from Gabon (Imboumy-Limoukou et al., 2016) where higher antibody responses were found in rural children compared to those in the urban areas.

Though the larger area of Hohoe municipality and its environs is designated as having medium but seasonal malaria transmission (Afari et al., 1992; Kweku et al., 2015; Kyei-Baafour et al., 2021), the micro-ecological and transmission differences and practices between the rural and urban settlements in the municipality may result in different vector exposure rates. The overall parasite prevalence was 7.3% by microscopy and 13.5% by PCR. Our data indicate that *P. falciparum* was more prevalent in the rural settlements compared to the urban settlement confirming previous reports (Robert et al., 2003; Lourenço, 2012). The high parasite prevalence in the rural settlements is indicative of higher transmission at those sites compared to the urban centre. The population in the rural communities mostly engage in agrarian activities with rice and vegetables being the main crops farmed. The water used in the rice farms could potentially serve as breeding sites for malaria vectors, thereby increasing the parasite exposure of people living in rural areas. Also, practices in rural settlements such as storing water in open receptacles, coupled with the bushes around could be good breeding grounds for the vectors (Azabre et al., 2013). Other practices in many rural settlements in Ghana likely to increase the risk of malaria are the habit of sleeping outside, especially in the dry season, and early evening and morning activities (Monroe et al., 2015). On the other hand, the urban centres have less favourable breeding grounds for vectors due to improved infrastructure and easy access to health care (Lindsay et al., 1990; Alirol et al., 2011). That notwithstanding, urbanization without the proper infrastructure may increase vector breeding sites and increase transmission as is recently reported (Mzilahowa et al., 2016).

We found higher total IgG responses to all the three antigens in the rural settlements compared to the urban settlements. This was confirmed by the higher seroprevalence (the proportion of children that responded to each antigen) in the rural settlements. This higher response may be due to the high parasite prevalence in rural communities. Also, parasitaemic children in the rural communities had higher total IgG responses to MSP2-3D7 and CSP, while no differences were observed in the urban settlements between parasitaemic and non-parasitaemic children. Parasite specific antibody responses can be boosted by *Plasmodium* infection by an estimated 20% increase in level (Kaddumukasa et al., 2015) and thus the high responses observed in the rural settlements are likely to reflect the higher parasite prevalence there. The low responses and low seroprevalence in the urban settlement may be due to the awareness of the population about malaria-related illness and protecting children from infection risk. Also, some of the practices in the villages, such as storing water in open receptacles, are hardly observed in the urban communities, which are served by piped water. A study comparing antibody responses between older Gambians and younger Ghanaians suggest that the younger children are unable to produce long-lived IgG antibody secreting cells compared to their older counterparts (White et al., 2014). Thus, children with intermitnent infection such as those in the rural community, are more likely to bost and sustain antibody production comapared to those less exposed (Fowkes et al., 2012). This may explain the higher responses in the rural children compared to those in the urban area. There are reports of antibody half-lives to different antigens (Ondigo et al., 2014) where some responses were found to long-lived. This long-lived responses may be a limitation to the use of such antigens to monitor malaria transmission intensity.

In an age-related analysis, high antibody responses, and seroprevalence in rural area in older children supports previous report of age-related antibody acquisition (Stanisic et al., 2015). Though there was no difference in the ages of the children from both settlements, higher seroprevalence associated with age in the rural settlement may be an indication of higher vector exposure. Interestingly, seroprevalence to MSP2-FC27 in the younger children was high in the urban area and the reason for this is not readily understood. Although Hohoe is an urban settlement, it is also a commercial town, and women are mostly involved in trading. This exposes the younger children who are carried at the back by their mothers to early morning bites when the mothers go out to the market at dawn. Though we do not have data on the specific strains of circulating parasites in our study area, which is a limitation, the specificity of the seroprevalence to MSP2-FC27 in the younger children may be an indication this parasite clone may be the predominant clone in the urban settlement.

In a logistic regression model adjusting for confounders, we found higher odds of responses to MSP2-3D7 to be associated with parasite carriage and thus higher transmission in the rural settlement which may indicate its circulation in the rural settlement. Serology, which was the tool employed in this study has confirmed that in an area of low to medium malaria transmission, micro-differences in transmission can be detected using anti-malaria antibody responses (Kusi et al., 2014; Kusi et al., 2016). However, the use of some of these anti-malaria antibody responses such as CSP to monitor transmission intensity and pattern in the future may be challenging with the introduction of RTS,S vaccine. This is because the RTS,S vaccine has CSP as the main component (Cohen et al., 2010; Laurens, 2020), and responses to the vaccine induced CSP may dilute naturally acquired responses. Thus, the need to increase the repertoire of antigens.

In summary, the study highlights heterogeneity in malaria transmission at the micro level and also confirms serology as a robust tool for transmission monitoring, especially in low to medium transmission settings. This has implications for malaria control and the administration of vaccines in such communities.

## Data availability statement

The original contributions presented in the study are included in the article/supplementary material. Further inquiries can be directed to the corresponding author.

## Ethics statement

The studies involving human participants were reviewed and approved by The Research Ethics Committee of the University of Health and Allied Sciences (UHAS-REC A.1 (5) 18–19). Written informed consent to participate in this study was provided by the parent/legal guardian.

## Author contributions

Conceived and designed the experiments: EK-B, LH, MK and MFO. Performed the experiments: EK-B, MO, AF, BAc, EAO. Analyzed the data: EK-B, KK, and MFO. Contributed reagents/materials/analysis tools: KK, BA, and MT. Wrote the manuscript: EK-B, KK, BA, LH and MFO. All authors contributed to the article and approved the submitted version.
